# *Trypanosoma dionisii* in China: ecology and tentative epidemiology

**DOI:** 10.1186/s40249-025-01336-2

**Published:** 2025-07-07

**Authors:** Qin Liu, Mu-xin Chen, Yu-chun Cai, Yuan-yuan Li, Zi-yi Wang, Yun-hai Guo, Yu-wan Hao, Jing-bo Xue, Yi-dan Jing, Fan-na Wei, Yong-bin Wang, Yue-jin Li, Hai-fang Wang, Jun-ling Sun, Ya-li Wang, Gang Wang, Na Wang, Nai-li Guo, Jian-cun Fang, Wei-xiao Chen, Xun-ming Zhou, Yang Yu, Yi Zhang, Jun-hu Chen, Qiang Wang, Shi-zhu Li, Ge Yan, Qun Li

**Affiliations:** 1 National Key Laboratory of Intelligent Tracking and Forecasting for Infectious Diseases; National Institute of Parasitic Diseases, Chinese Center for Disease Control and Prevention; Chinese Center for Tropical Diseases Research; NHC Key Laboratory of Parasite and Vector Biology; WHO Collaborating Centre for Tropical Diseases; National Center for International Research on Tropical Diseases, Ministry of Science and Technology, Shanghai, 20025 China; 2 Shandong Institute of Parasitic Disease, Shandong First Medical University (Shandong Academy of Medical Sciences), Jining, 272033 Shandong China; 3 National Key Laboratory of Intelligent Tracking and Forecasting for Infectious Diseases, Chinese Center for Disease Control and Prevention, Beijing, 102299 China; 4 Department of Infectious Disease, Qilu Hospital, Cheeloo College of Medicine, Shandong University, Jinan, 250012 Shandong China; 5 Disease Control and Prevention Center of Dongying City, Dongying, 257092 Shandong China; 6 Key Laboratory of Animal Ecology and Conservation Biology, Institute of Zoology, Chinese Academy of Sciences, Beijing, 100101 China; 7 School of Life Sciences, University of Science and Technology of China, Hefei, 230026 Anhui China

**Keywords:** Human trypanosomiasis, *Trypanosoma dionisii*, *Trypanosoma vespertilionis*, Co-infection, Bats, Companion animals, China

## Abstract

**Background:**

*Trypanosoma dionisii*, one of several species that parasitizes Chiroptera worldwide, was first reported in a 30 year-old pregnant woman in China. It is important to improve our understanding of ecological and epidemiological patterns to identify potential transmission vectors and to estimate the risk of *T. dionisii* infection in the local population as well as in various species of domestic and wild animals.

**Methods:**

We performed an ecological survey with epidemiological features in the area where the first *T. dionisii* case was found, including parasitological and serological tests and local demographic information for six surrounding villages. Sylvatic and domestic mammals and potential vector organisms in the same locality were investigated by nested-PCR for *Trypanosoma* and the phylogenetic analysis was performed.

**Results:**

A total of 241 samples from the local population were screened for trypanosomiasis by parasitological and serological tests with no positive cases identified. However, 11 out of 18 bats collected from the village tested positively for *Trypanosoma* spp. by microscopy and nested-PCR, while 9 were positive for *T. dionisii* and 2 for *T. vespertilionis*. With regard to cats, 5 from a pet hospital in local showed 3 were co-infected with of *T. dionisii* and *T. vespertilionis,* and one having *T. dionisii* only, as well as one of the 29 animals examined was found infected with *T. vespertilionis*. Other animals seemed even less affected as all 163 blood samples collected from livestock and poultry, such as cows, sheep, chickens, ducks and geese, tested negative. Also 35 mosquito and mite pools tested negatively, while 4 out of 30 tick pools tested positive by nested-PCR with their sequences close to *T. conorhini*.

**Conclusions:**

The survey indicates that a natural epidemic foci of *T. dionisii*, exists in Dongying Region, Shandong Province, China. Although no evidence of a high risk for human epidemic was found, the widespread presence of this parasites in bat species and a relatively high infection rate observed in the surveyed cats and dogs emphasize the emerging threat it poses to human health. Further surveillance and analysis are warranted to evaluate the transmission risk.

**Graphical Abstract:**

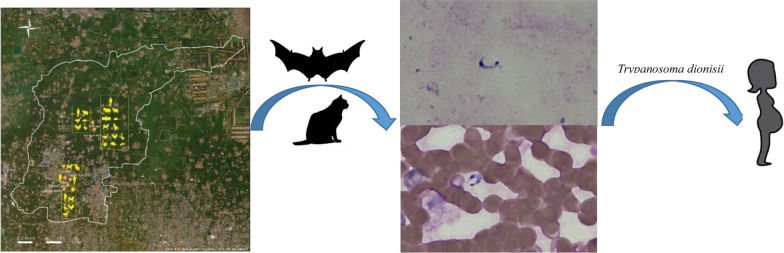

## Background

The trypanosomes are kinetoplastid hemoflagellate parasites belonging to the genus *Trypanosoma*, family Trypanosomatidae with a broad host range transmitted mainly by several different invertebrates through horizontal and vertical routes [1–3]. These parasites are widespread globally and can infect humans and various animal species. The *Trypanosoma* genus of mammalian parasites comprises flagellate species of seven subgenera; three stercorarians and four salivarians [4, 5]. Human infections caused by trypanosomes include African trypanosomiasis (sleeping sickness) and American trypanosomiasis (Chagas), the former caused by *Trypanosoma brucei gambiense* and *T. b. rhodesiense*, and the latter by *T. cruzi.* The tsetse fly is the vector in equatorial Africa, with triatomine insects playing this role in Latin America [6–8]. African trypanosomiasis has 100% mortality if left untreated, while the American kind is less acute, but still leads to high morbidity and mortality. Apart from these main trypanosomes, atypical human species, such as *T. evansi*, *T. lewisi*, *T. lewisi*-like and *T. congolense* have been reported [2, 9–13]. This raises the question whether they have the potential to evolve as important new pathogens for humans or whether they are simply opportunists [2, 14].

*T. dionisii* is closely related to *T. cruzi* [15, 16]. The data indicate that metacyclic forms of *T. dionisii* and *T. cruzi* share characteristics, e.g., with regard to epimastigote epitopes [17] and the ability to enter non-phagocytic human cells in a manner dependent on lysosome exocytosis [16]. However, in contrast to *T. cruzi*, *T. dionisii* has been found to penetrate cell nuclei [16, 17]. *T. dionisii* has been detected in human cardiac tissue together with four of the seven discrete typing units (DTUs) of *T. cruzi* (TcI-IV) in a 2 year-old patient who had acquired acute Chagas disease [18]. The patient subsequently died, and a recently dead but infected reduviid bug (*Triatoma vitticeps*) was believed to be the origin of this *T. cruzi* infection [18]. Although *T. dionisii* is considered non-pathogenic, experimental studies by Oliveira et al. have reported the replication of *T. dionisii* in mammalian cells in vitro [19]. Additionally, Gardner and Molyneux found *T. dionisii* amastigote stages in thoracic skeletal muscle from *Pipistrellus pipistrellus* [20]. Collectively, these findings suggest *T. dionisii* may cause adverse effects in mammalian hosts. Further investigations of the clinical impact of *T. dionisii* are warranted, especially given its global distribution and that it recently was shown to lack bat host specificity [18, 20–22].

Recently, a 30 year-old, pregnant woman with persistent fever up to 39.5 °C, mild myocarditis and splenomegaly, was diagnosed as *T. dionisii* infection in Qilu hospital, Shandong Province, China [23]. The current study was initiated in late April 2024 to investigate the possible sources of infection and transmission routes, and to assess the risk of spread. It had the following objectives: (i) to investigate whether some individuals in the population in Guangrao County had latent trypanosomiasis, and (ii) to study the ecology of the transmission cycle of *T. dionisii* in the area where the *T. dionisii* infection occurred and in nearby locations. The overall aim of these objectives was to evaluate the risk of this disease in human populations and contribute to the development of local control measures to prevent additional cases of *T. dionisii* infection.

## Methods

### Study area

Guangrao County belongs to Dongying City, Shandong Province and has a total area of 1166 km^2^, 553 villages, a permanent population of 525,000 and an urbanization rate of 61.7% [24]. The area where the *T. dionisii* case occurred is characterized as an area of plains with many factories and a strong economic foundation. Many residents of the surrounding rural area have moved out and some houses there are vacant.

The study was conducted at six localities in this area: four residential communities in Guangrao Street, Guangrao County (i.e. East Hupan New City, West Hupan New City, Guoan Community, Gongshou Liu Village), which are all located near the place where the first *T. dionisii* case occurred; two villages in Daozhuang Town (i.e. Xiang Zhuang Village and Jijiatuan Village) where the parents of this *T. dionisii* case live; and a lake park named Sunwuhu near the residential communities.

### Human population screening

Blood samples were collected from permanent Guangrao County residents during April 22 to 24, 2024, including the first *T. dionisii* case and parasitological, serological and molecular tests were subsequently performed. Collection focused on people with various clinical symptoms, such as fever of unknown origin, hepatosplenomegaly, emaciation or anorexia, but also included random residents of the five medical institutions of Guangrao County and six localities. Additional samples were collected from September 2023 to April 2024 from patients in neighbourhood community hospitals diagnosed with myocarditis, lymphadenitis, esophagus enlargement or megacolon. All samples were investigated by rapid detection test (RDT) and Enzyme-Linked Immunosorbent Assay (ELISA).

### Samples from small, wild mammals

Field work was conducted during April 22 to 26, 2024, a few months after the occurrence of the first *T. dionisii*-infection case. Small wild mammals were captured using the following protocol: aiming to trap animals alive, linear transects were designed with capture points 100 m apart using Sherman cages baited with a mixture of deep-fried dough sticks for rodent animals [18] and mist nets for bats [25]. The traps were placed near houses and also in wild animal habitats in various types of environment. Seven transects with 10 traps were centred on Guangrao Street, Guangrao County, Dongying City, Shandong Province, China. Bats were also sampled from the eaves of farmers’ houses. A total of 23 animals were captured and blood samples were collected by puncturing the cephalic vein under aseptic conditions, siphoning off about 0.2 ml into Vacutainer^®^ tubes with anticoagulant and taken to the laboratory. Parasitological and molecular tests were performed on each sample.

### Domestic animals survey

An active search for domestic animals (including cattle, sheep, dogs, cats, chickens, ducks, geese, and other livestock) was performed during April 22 to 26, 2024 at the following locations: Xiang Zhuang Village and Jijiatuan Village, and the pet hospital in Hupan New City Community. Blood samples from domestic animals at all of these locations were collected (with the owners’ consent) in Vacutainer^®^ tubes as described for the wild animals. Questionnaires were given to the owners requesting the following information: age, sex and name of the animal as well as its feeding and management status (free-range or captive breeding). Each animal was considered separately, even if they were from the same house or the same hospital. A total of 168 animal samples were collected and the characteristics of breeding environments were recorded. Parasitological and molecular tests were performed on each sample.

### Vector survey

In order to detect various potential vectors, surveys were conducted at the same time in the six localities. Mosquito collection was carried out in livestock sheds, homes and in the field by lamp trapping. Ticks and mites on wild and domestic animals were collected as well as triatomines and bedbugs in various environments. Different insects were included in the surveys although the presence of trypanosomes in all of them has not been reported before. Molecular tests were the only diagnostic approach performed on the vectors.

The vector samples collected were classified and identified according to morphology, and kept frozen in storage tubes for further *Trypanosoma* detection by PCR with collection time, place and species recorded. They were distributed between tubes as follows: 2–6 mosquitoes/tube, 1 adult tick/tube, 5 nymph ticks/tube and 4–5 mites/tube.

### Investigations

#### Direct testing

Thick and thin blood smears from fresh blood samples were prepared a Giemsa-stained examination was conducted to visualize potential *Trypanosome* flagellates. Two stains were conducted for each sample.

#### Serology

A serological survey for the detection of anti-*T. cruzi* IgG antibodies in the human population was performed using a rapid diagnostic test (RDT) (InBios, Seattle, WA, USA) as described by Whitman et al. [26]. RDT-positive samples were rechecked using a *T. cruzi* ELISA IgG + IgM kit (Vircell, Granada, Spain) as described by Suescun-Carrero et al. in 2021[27].

#### Molecular diagnostics

Molecular characterization tests were conducted by extracting Deoxyribonucleic Acid (DNA) from the collected blood and vector organisms using the QIAamp DNA Mini Kit (Qiagen, Hilden, Germany). A nested PolymeraseChainReaction (PCR) strategy for detecting and identifying *Trypanosoma* species as described by Noyes et al. [28] was used. External primer TRY927F 5′-CAGAAACGAAACACGGGAG-3′and TRY927R5′-CCTACTGGGCAGCTTGGA-3′ and internal primers SSU561F 5′-TGGGATAACAAAGGAGCA-3′ and SSU561R 5′-CTGAGACTGTAACCTCAAAGC-3′) were used under PCR amplification conditions: 94 °C for 3 min; 30 cycles at 94 °C for 30 s, 55 °C for 60 s, 72 °C for 90 s; and 72 °C for 10 min [28]. All of the samples were sequenced for both DNA strands with the BigDye Terminator v. 3.1 cycle sequencing kit (Applied Biosystems, Foster City, CA, USA) on an ABI 3730 DNA sequencer available from Sangon Biotech (Shanghai, China). The amplicons were purified using the MiniBEST Agarose Gel DNA Extraction Kit (Takara, Dalian, China) according to the manufacturer’s recommendations and subsequently ligated into the PMD20-T vector (Takara, Dalian, China). Ten positive clones were sent to Sangon Biotech for sequencing of both strands. The sequencing reactions were performed using the same sequencing kit with Rimers M13 in an ABI 3730 sequencer.

### Implementation and phylogenetic analysis

The sequences were edited, aligned and corrected using the BioEdit software (https://bioedit.software.informer.com/). The sequences were compared with nucleotide sequences deposited in GenBank using the Basic Local Alignment Search Tool (BLAST). Phylogenetic tree construction was performed using MEGA version 13 [29]. We used the maximum likelihood method, employing a DNA substitution model. The best substitution model was identified as having the lowest Bayesian Information Criterion score: Kimura two-parameter [30] for small subunit ribosomal ribonucleic acid (SSU rRNA), with bootstrapping at 1000 replications.

The sequences of *T. cruzi* calde including *T. dionisii* (LC326397.1 from Japan, MK625434.1, MH393942.1 and PP177438.1 from China, MZ606796.1 from Thanland, MN956693.1 from Switzerland, FJ001667.2 from Brazil, OR660627.1 from Mexico, OR597300.1 from Russia, MT533286.1 from Australia and AJ009151.1from UK), *T. vespertilionis* (AJ009166.1 and MF144888.1), *T. conorhini* (XR_003828665.1 and AJ012411.1), *T. livingstonei* (KF192984.1), *T. erneyi* (JN040989.1), *T. cruzi* (FJ001664.2, AY785561.1 and FJ900239.2), *T. noyesi* (KX008320.1), *T. rangeli* (XR_003828669.1) and *T. minasense* (,AJ012413.1) were extracted from GenBank as references. *Parabodo caudatus* (DQ207590.1) was used as the outgroup. All sequences analyses were deposited in the GenBank database under their accession numbers.

## Results

### Human population screening

#### Case follow-up

The first *T. dionisii*-infection case [23] was treated with Benznidazole December 2023 and tested negative by RDT and ELISA (ELISA value 4.34) when followed-up on April 22, 2024. The parasite was absent in blood smears and the nested-PCR test was also negative. When tested before treatment, the patient was positive (ELISA value 19.62), which was 4.52 times higher than the ELISA value measured in plasma collected this time (4 months after treatment).

#### Population screening

A total of 241 blood samples were collected, including 49 blood samples from patients with unexplained fever or myocarditis treated in 12 medical institutions in Guangrao County since September 2023, 130 samples from key populations collected from 6 villages (residences), and 62 randomly selected individuals from the hospital's physical examination population.

The 49 blood samples from the patients with unexplained fever or myocarditis from 12 medical institutions in Guangrao County were all negative by RDT and ELISA, as were the 62 blood samples from 5 medical institutions in Guangrao County. No trypanosomes were detected in the blood smears and the nested-PCR tests were also negative.

Although three of the 130 blood samples from key populations collected from the 6 localities in the study area were found to be positive by RDT, they were negative by ELISA, and no trypanosomes were detected in the blood smears and the nested-PCR results were also negative. Summary results are shown in Table 1.

**Table 1:** Human population screening results in study areas

Population category	Location	Sample	RDT	Blood smear microscopy	18S rRNA nested-PCR	ELISA*	Conclusion
Fever/myocarditis	Guangrao County	49	Neg	Neg	Neg	Neg	Neg
Surrounding population of the first case residence area	Hupan New City (two communities)	42	Neg	Neg	Neg	Neg	Neg
	Guoan Community	20	1 (+)	Neg	Neg	Neg	Neg
	Gongshou Liu Village	20	2 (+)	Neg	Neg	Neg	Neg
	Xiangzhuang Village	28	Neg	Neg	Neg	Neg	Neg
	Jijiatuan Village	20	Neg	Neg	Neg	Neg	Neg
Medical institutions In Guangrao County**	People's Hospital of Guangrao County	10	Neg	Neg	Neg	Neg	Neg
	Guangrao County Traditional ChineseHospital	20	Neg	Neg	Neg	Neg	Neg
	Maternal and Child Health Guangrao County Hospital	10	Neg	Neg	Neg	Neg	Neg
	Second People's Hospital of Dongying city	12	Neg	Neg	Neg	Neg	Neg
	Street Health care Center of Guangrao County	10	Neg	Neg	Neg	Neg	Neg
Total		241	3 (+)	0	0	0	0

^*^by *T. cruzi* ELISA IgG + IgM kit

^**^Randomly sampled people in Guangrao County who were neighbours of first *T. dionisii* case. Neg, Negative

### Wild animals

#### Bats

**Fig. 1:**
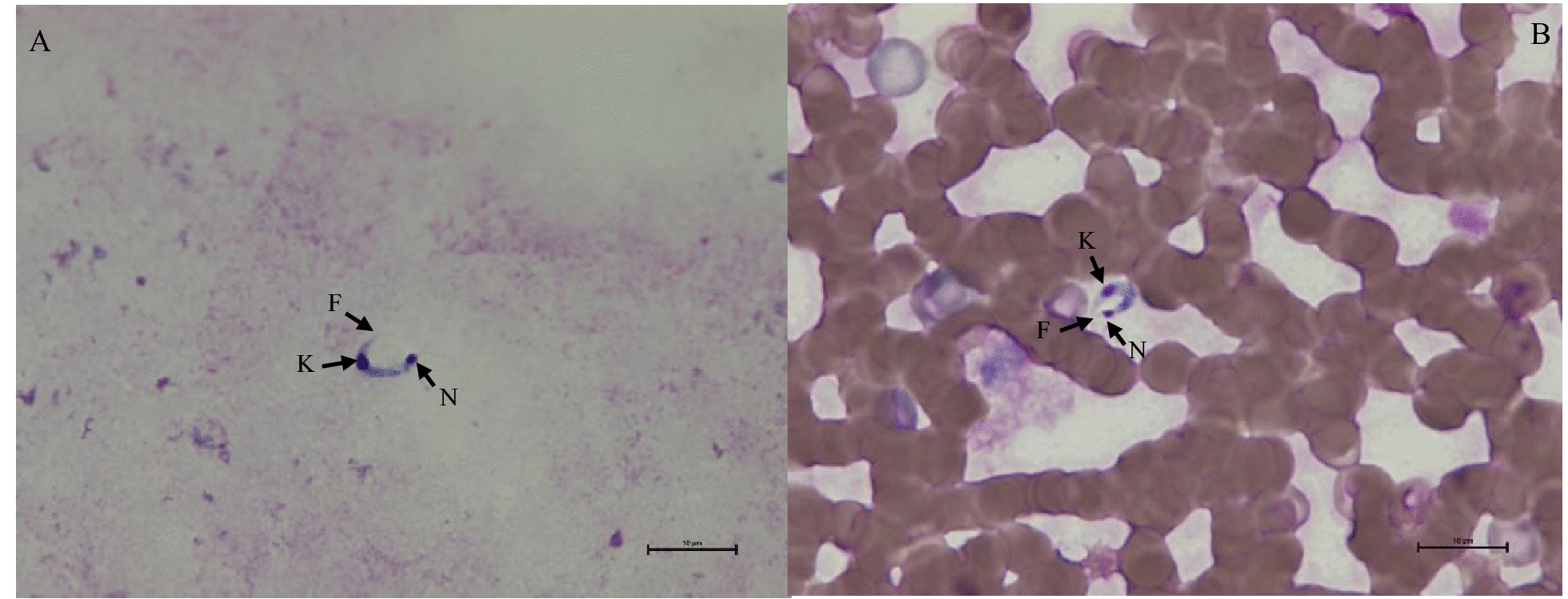
The morphology of *T. dionisii* in bat blood. **A** thick smear; **B** thin smear; F: free flagellum; K: kinetoplast, N: nucleus

Eighteen bats were captured in Xiangzhuang Village, including 6 *Hypsugo alaschanicus*, 6 *Pipistrellus abramus* and 6 *Eptesicus serotinus*. *Trypanosoma* spp*.* were found in 5 (27.8%) of them by blood smear microscopy (Table 2). Four were detected in *H. alaschanicus* and one in *E. serotinus*. Typical epimastigote features, such as the presence of a nucleus, free flagellum and kinetoplast were observed under the microscope (Fig. 1). The epimastigote is elongated and oval, with the nucleus located near the middle and rear part of the body. The unique, circular, large kinetoplast of *Trypanosoma* spp. is prominently located at the posterior end of the body (Fig. 1).

**Table 2:** Positive bat samples detected by microscopic examination and nested-PCR

Number	Experiment (no.)	Bat species	18S rRNA nested -PCR and sequence	Microscopic examination
1	20240422-01	*H. alaschanicus*	*T. dionisii*	Positive
2	20240422-02	*H. alaschanicus*	*T. dionisii*	Positive
3	20240422-05	*P. abramus*	*T. vespertilionis*	No detected
4	20240424-02	*H. alaschanicus*	*T. dionisii*	Positive
5	20240424-03	*E. serotinus*	*T. dionisii*	No detected
6	20240424-04	*E. serotinus*	*T. dionisii*	Positive
7	20240424-05	*P. abramus*	*T. dionisii*	No detected
8	20240424-06	*P. abramus*	*T. vespertilionis*	No detected
9	20240425-04	*H. alaschanicus*	*T. dionisii*	Positive
10	20240425-05	*E. serotinus*	*T. dionisii*	No detected
11	20240425-06	*E. serotinus*	*T. dionisii*	No detected

**Fig. 2:**
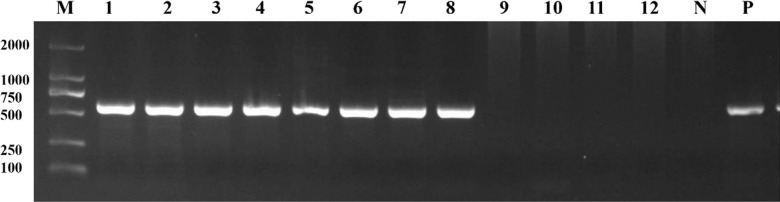
Nested-PCR results of the 12 bat blood DNA samples. DNA samples 1 to 8 were positive with target bands of about 600 bp, while bands No. 9 to 12 were negative

Eleven out of the 18 bat blood samples were amplified with target bands using nested-PCR and valid sequences were obtained for all of them through sequencing (partial amplification results are shown in Fig. 2). After comparison with GenBank sequences, the results showed that 9 sequences were *T. dionisii* with a similarity of 99.2–99.6% and two were *T. vespertilionis* with a similarity of 99.4–99.6%. The molecular detection rate was 61.1% (11/18), as shown in Table 2. Of the 9 T*. dionisii* specimens, 4 were from *H. alaschanicus,* another 4 were from *E. serotinus,* one was from *P. abramus,* while the 2 T*. vespertilionis* were from *P. abramus*. The infection rate of *T. dionissi* was 66.7% (4/6) in both *H. alaschanicus* and *E. serotinus*. In *P. abramus*, the infection rates of *T. dionissi* and *T. vespertilionis* were 16.7% (1/6) and 33.3% (2/6), respectively. All the samples positive by microscopic examination samples were also positive by PCR and all of them were found to be *T. dionissi*.

#### Rats

Three of the five wild rats captured in the neighbourhood of Jijiatuan Village were *Rattus flavipectus* and two were *R. norvegicus*. No *Trypanosoma* spp. was detected in these rats; neither by microscopy nor nested-PCR.

### Domestic animals

Blood samples from a total of 134 domestic animals were collected from rural villages. These samples included 4 cows, 29 sheep, 73 chickens, 11 ducks and 17 geese, all of which tested negative for *Trypanosoma* by microscopy and nested-PCR.

Blood samples from pets, which included 29 dogs and 5 cats, collected from Hupan New City Community pet hospital, showed positive results for one dog and 4 cats by nested PCR corresponding to an infection rate of 3.4% for the dogs and 80.0% for the cats, while the microscopy of blood smears was negative for all these samples (Table 3). The positive sequences obtained were subjected to BLAST, with the results for the dogs showing 99.8% identity with *T. vespertilionis* isolates P14 and EU (AJ009166.1), and the results for three cats showing a mixed *T. dionisii/T. vespertilionis* infection, with 99.5–99.7% identity of *T. dionisii* 558CT and MPM (JPN) (FJ001667.2, LC326397.1) and 98.0–99.8% identity of *T. vespertilionis* isolates P14 and EU (AJ009166.1), respectively. The sample sequence from the remaining cat showed 99.8% identity of *T. dionisii* isolates 558CT and MPM (JPN) (FJ001667.2, LC326397.1).

**Table 3:** Overview of the diagnostic approaches of pets including those at least positive with one of the tests performed

No	Exp.(no.)	Species	Location	18S rRNA nested-PCR and sequence	Microscopic examination
1	G505	Chinese rural dog	Gongshou Liu Village	*T. vespertilionis*	No detected
2	M501	Calica cat	Hupan New City Community Hospital	Mixed infection*	No detected
3	M502	Blue cat string	Hupan New City Community Hospital	Mixed infection*	No detected
4	M503	British Shorthair Cat	Hupan New City Community Hospital	*T. dionisii*	No detected
5	M504	British Shorthair Cat	Hupan New City Community Hospital	Mixed infection*	No detected

^*^T. dionisii and *T. vespertilionis*

### Vectors

Twenty-six mosquitoes were captured in the six localities. The catch included 13 *Culex pipienspallens*, 6 *C. pipiens*, 7 *Anopheles sinensis*, which were all negative by PCR.

With regard to ticks, we caught a total of 94, including 81 collected by artificial banner laying and 13 from the bodies of the animals. Four tick samples (13.3%) had *Trypanosoma* spp. based on the SSU rRNA as shown in Table 4. Among the four positive ticks, Sdgr-16, Sdgr-23 and Sdgr-28 showed 100, 93.5 and 95.6% identity to *T. conorhini* (Tco025E_06706), while Sdgr-11 had 717 bp, which was nearly 100 bp longer than the others, showed only 79.9% identity to *T. conorhini* (Tco025E_06706) (Table 4).

**Table 4:** Characteristics of the four positive tick

No	Identity	Survey site	Parasiticanimal /Environment	Species	Tick quantity	Molecular detection
1	Sdgr-11	Xiang Zhuang Village	Dog	*Rhipicephalus turanicus* (adult)	1	Trypanosoma spp.
2	Sdgr-16	Sunwu Lake Woods	Deciduous forest	*Haemaphysalis flava* (adult)	1	*T. conorhini*
3	Sdgr-23	Sunwu Lake Woods	Deciduous forest	*H. longicornis* (nymph)	4	*T. conorhini*-like
4	Sdgr-28	East Woods of Lakeside New City	Grassland	*H. longicornis* (nymph)	5	*T. conorhini*-like

Nine mites were collected from the surface of bat body, including two species identified as Macronyssidae and Steatonyssus by morphology. The nested-PCR results for these were negative. No bedbugs or triatomine bugs were collected in this area.

### Phylogenetic tree

**Fig. 3:**
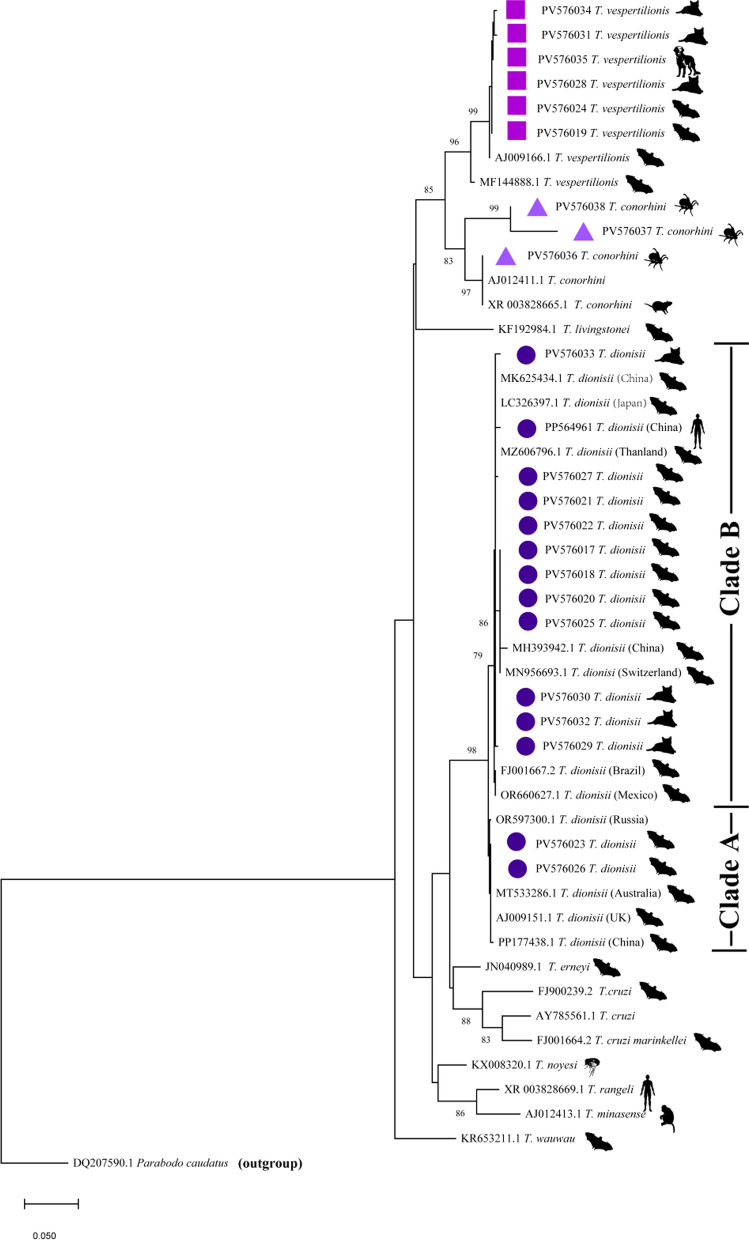
Molecular phylogenetic tree based on 600 bp SSU rRNA gene sequences. Maximum likelihood with bootstrapping at 1000 replications. Branch lengths were indicated by the scale bar. *T. dionisii, T. vespertilionis* and *T. conorhini* detected in this study were labeled with round,square and triangle font, respectively. *Parabodo caudatus* was used as an outgroup

A phylogenetic tree was established by comparing valid sequences: eleven from bat, seven from cat, three from tick, one from dog and one from the *T. dionisii*-infection case described previously [23] against the GenBank database. The sequences generated in this study were submitted to GenBank under the accession numbers PV576017–PV576038. The sequences from the nine bat samples, four cat samples and the sample from the first *T. dionisii*-infection were all clustered in the same branch as those of *T. dionisii*, while the sequences from the two bat samples, three cat blood samples and the one dog sample belonged to the same branch as those of *T. vespertilionis*. The sequences from three tick samples were clustered in the same branch as those of *T. cornohini* (Fig. 3).

## Discussion

*T. dionisii* is prevalent throughout the world and is found in many different species of bats [22, 31–33]. A study in Japan reported a prevalence of 2.1% (2/94) in *M. fuliginosus* [21], while higher infection rates, ranging from 9.6–36.0%, have been reported in bats in the United Kingdom, the Czech Republic, Brazil and Australia, with particularly high frequencies in Brazil and Australia [18, 34, 35]. A low prevalence of 0.4% (1/227) of *T. dionisii* in *Rousettus leschenaultii* has been reported in south-western China, with higher levels in *M. pequinensis* (11.0%) and *E. serotinus* (16.0%) in northern China [25, 33].

In this study, a high rate of *Trypanosoma* spp. was found in bats, primarily *T. dionisii* (50.0%) but also *T. vespertilionis* (11.1%). Even higher rates of *T. dionisii* were found in *H. alaschanicus* and *E. serotinus*. In addition, all five positive *T. dionisii* cases detected by blood smear occurred in *H. alaschanicus* and *E. serotinus*. These findings indicate that these Chiroptera species are particularly susceptible to *T. dionisii*. *T. vespertilionis* was detected only in *P. abramus, while T. dionisii* was detected in all three bat species. Taken together, these results suggest that *T. dionisii* is particularly adaptable to bat species.It is worth noting that the infection rate of *T. dionisii* in this area is very high, especially in *H. alaschanicus* and *E. serotinus*. Whether the high infection rate in bats is related to overflow to infect human individuals and other animals is worth further attention.

The fact that *T. dionisii* was found to be common in *H. alaschanicus* and *E. serotinus* in the study area indicates that it is a natural *T. dionisii* hotspot. The fact that bats tend to live in close contact with humans, at least in this area, favours human infection with *T. dionisii* there. During our investigation, we observed that bats live under the eaves of homes in this region, and *E. serotinus* bats were sampled from the eaves of farmers’ houses.

Importantly, this is the first time that *T. dionisii* has been reported in companion animals, which reinforces the idea that this parasite is probably more generalist than previously recognized. Apart from bat species, *T. dionisii* has been reported in various small mammals, particularly in rural areas [36, 37]. In our investigation, the detection rate was particularly high in cats, where we found prevalence to be as high as 80.0% (4/5), though it should be admitted that the number of cats investigated was comparatively low. The situation with regard to dogs was the opposite, as we detected only one trypanosome (*T. vespertilionis*) in one dog out of 29 investigated. Interestingly, the consistency of trypanosome species in bats as well as in dogs and cats suggests that companion animals might be as suitable hosts for trypanosomes as bats are, at least for *T. dionisii* and *T. vespertilionis.* In addition, the results presented here indicate a sign of the possibility of transmission between animals, something which undoubtedly increases the risk of human infection with these trypanosome species.

The biological vectors for *T. dionisii* are perhaps members of the family Cimicidae, a group of blood-sucking insects (vectorial transmission) with a worldwide distribution, most of which are biologically and ecologically associated with bats [20, 36, 38, 39]. However, the vector of *T. dionisii* is still unknown. There are no reports of Cimicidae in the study area, which highlights our limited knowledge about these ectoparasites. A recent report argues that the gamasine mite *Steatonyssus periblepharus* may be a vector of the “clade A” of *T. dionisii* [40]. The literature maintains that triatomine bugs, bedbugs, tsetse flies, fleas, mites, mosquitoes, and ticks are the main vectors of *Trypanosoma* spp*.* [41–46]. In this investigation, we checked for trypanosomes in all possible potential vectors that could be collected and found that *T. cornohini*, *T. cornohini-like* spp can infect *H. flava*, *H. longicornis* and *R. turanicus* (Table 4). These vectors exist in this area and can carry various species of trypanosomes; thus it might be possible that they could be the vector transmitting *T. dionisii*. *T. cornohini* has been confirmed to be transmitted by *Triatoma rubrofasciata* [5, 47]. The fact that *T. cornohini* DNA was detected in ticks for the first time is important and indicates that the vectors of trypanosomes are diverse. Previously, several studies on* Trypanosoma *in ticks have been conducted across various regions, leading to the discovery and description of trypanosome species from their tick hosts [48]. The authors concluded that tick species were vector candidates for trypanosome species. For example, the ixodid tick was identified as a hypothetical vectorial candidate of *T. copemani* and *T. vegrandis* [49]. The usual definition of *T. conorhini* as a parasite of *Rattus rattus* transmitted by *T. rubrofasciata*. However, in this region, no triatomines were detected. Whether there is a new circulation pathway for *T. conorhini*, which needs further study in the future. The bat mites collected from the body of the bats have reportedly been suspected as potential biological vectors for *T. dionisii* [40], but we found no *Trypanosoma* spp. Knowledge of the transmission of *T. dionisii* is still limited and it is urgent to determine whether mites can be involved in the transmission cycle of *T. dionisii*, especially in the study region, where a human case has recently been recorded [23]. The role of companion animals found in the transmission cycle of *T. dionisii* in this area is of interest in this connection. These descriptions demonstrate that very little information is available about the dynamics of the transmission cycle of this parasite in nature, and the hosts and vectors involved. Further investigation on a large-scale population surveillance based on more sensitive and specific detection methods was needed, as well as animal and vector surveys to assess the risk of this disease in this region, or even in Shandong province. Meanwhile, given its potential to infect mammalian species other than bats, understanding its pathogenesis to humans and the mode of transmission is imperative.

For the first time, the presence of *T. dionisii* has been shown in bats and companion animals in China. The sequence from the first *T. dionisii*-infection case [23], the cats and most of the bats were found to be close to the sequences of *T. dionisii* found in bats from Japan (LC326397.1) and China-Yunnan (MK625434.1) in clade B in the phylogenetic tree suggesting a local infection. Meanwhile, *T. dionisii* (PV576023 and PV576026) from two bats were close to *T. dionisii* found in bats from Russia (OR597300.1) and Australia (MT533286.1) in cladeA (Fig. 3). It is interesting that two subgroups of *T. dionisii* were detected in the same place in different species of bats. Whether different subpopulations of *T. dionisii* circulate there need further attention.

Molecular diagnostics was useful for the detection of co-infection. Mixed infections by two or three DTUs of *T. cruzi* in free-living wild mammals and human beings have been described [50, 51] and mixed infection by four DTUs of *T. cruzi* and *T. dionisii* has also been described [18]. Our study reinforces the importance of the molecular detection for *Trypanosoma* spp. The occurrence of natural, mixed infections is frequent, yet the implication for host and disease dynamics remains poorly understood. It has been suggested that mixed infections could modulate host immune responses and potentially enhance pathogenicity [30], but this has not been substantiated. Mixed infections may arise from repeated exposure to various parasite genotypes and species via different routes and at varying intervals [14]. Whether mixed infections are worse than a single infection requires further study and emphasizes the need for development of targeted preventive and control strategies to safeguard the public. 

## Limitations

It is crucial to acknowledge the limitations of this study. Although we have confirmed that local wild and companion animals are infected with *T. dionisii*, as well as which constitutes a natural epidemic foci of *T. dionisii*, the transmission route is still unclear. We have investigated some vector organisms, but no *T. dionisii* gene fragment was detected. The number of potential vectors and domestic animals (especially cats and dogs) was limited in this survey, and it was also worth whether there was seasonal bias. The more sufficiently sensitive detection method should be used and further efforts are needed to strengthen monitoring activities. In addition, a larger number of vectors and companion animals would have added strength to the study. 

## Conclusions

Our research shows that bats, companion animals and ticks can carry infections by *T. dionisii*, *T. vespertilionis* and *T. conorhini* and that the Guangrao area in Dongying, Shandong Province, China could be a natural epidemic foci of *T. dionisii.* This result expands the distribution of these *Trypanosoma* spp. in this province. Not only bats, but also some pets can thus be regarded as reservoir hosts of trypanosomes, which carry a risk of local transmission between animals, with a potential to also include humans. Although other trypanosome species were detected in ticks in this area, the vector of *T. dionisii* is still unknown. This survey provides important information for the prevention and control of trypanosomiasis, and helps formulate targeted prevention and control measures.

## Data Availability

All data used in this paper has been presented.
